# Neonatal exposure to di-(2-ethylhexyl) phthalate (DEHP) through breastfeeding leads to dysfunction endocrine-metabolic outcomes in male rats at adulthood

**DOI:** 10.3389/fendo.2026.1826776

**Published:** 2026-06-03

**Authors:** Thayná Martins Macario, Anne R. Melo Santos, Natalia da Silva Lima, Leandro Miranda-Alves, Ana Paula Santos-Silva

**Affiliations:** 1 Núcleo Multidisciplinar de Pesquisa em Biologia (NUMPEX-Bio), Campus UFRJ - Duque de Caxias Prof. Geraldo Cidade, Universidade Federal do Rio de Janeiro, Duque de Caxias, Brazil; 2 Research Group on Endocrine Disruptors and Metabolic Programming, Universidade Federal do Rio de Janeiro, Duque de Caxias, RJ, Brazil; 3 Regulation of Gene Expression and Applications (EXPRELA) Group, Interdisciplinary Center of Chemistry and Biology, Universidade da Coruña, A Coruña, Spain; 4 Faculty of Sciences, Universidade da Coruña, A Coruña, Spain; 5 Universidade Federal do Rio de Janeiro Instituto de Ciencias Biomedicas, Rio de Janeiro, Brazil

**Keywords:** development, health, metabolism, obesity, pollutants

## Abstract

The increased production of industrial chemicals has led to greater human exposure to endocrine disruptors, such as diethylhexyl phthalate (DEHP). This plasticizer, widely used in hygiene products, packaging, and medical devices, does not covalently bind to polymeric materials, which facilitates its migration and subsequent entry into the human body, primarily via the oral route. In this study, we investigated the effects of DEHP exposure during the lactation period within the framework of the Developmental Origins of Health and Disease (DOHaD) concept. The experimental model utilized Wistar female rats (n=9) that were placed for mating. Litters were standardized to six male pups (n=6) each. DEHP was administered in dams via oral gavage during the lactation (post-natal day, PND 1-21) period to three groups (n=3 dams/group): vehicle, 100 mg/kg/day, and 500 mg/kg/day. After weaning, the offspring were maintained until adulthood.At PND 90, a total of nine male pups (n=3/litters/group) were randomly selected for euthanasia and analyses. At weaning (post-natal day, PND21), animals in the 500DEHP group exhibited reduced body weight and central adiposity, accompanied by increased serum insulin levels, an elevated β-cell function index (HOMA-β), and higher serum total triiodothyronine (T3) levels. By adulthood (PND90), the 500DEHP group developed a delayed obesogenic phenotype, characterized by hyperphagia and increased relative visceral white adipose tissue (vWAT) mass. This phenotype was associated with impaired leptin secretion by vWAT, resulting in reduced circulating leptin levels. Consequently, dysregulation of hypothalamic appetite control pathways was observed, marked by decreased expression of the anorexigenic neuropeptide proopiomelanocortin (POMC) and increased expression of the orexigenic neuropeptide neuropeptide Y (NPY), concomitant with reduced suppressor of cytokine signaling 3 (SOCS3) levels. Notably, elevated serum total T3 levels persisted into adulthood. In contrast, in the 100DEHP group, hypothalamic dysfunction appeared to be the primary target, evidenced by a simultaneous increase in both POMC and NPY expression. Collectively, these findings demonstrate that neonatal exposure to DEHP during lactation influences adult phenotype in a dose- dependent manner. Higher-dose exposure (500 mg/kg/day) compromises peripheral metabolic homeostasis, particularly through vWAT dysfunction and impaired leptin secretion, whereas lower-dose exposure (100 mg/kg/day) preferentially affects central regulation, suggesting dysregulation of hypothalamic neuropeptides.

## Introduction

Nowadays, the synthesis and use of industrial chemicals have become increasingly widespread, particularly to enhance production efficiency and improve the resistance and durability of materials. Some of these substances have been identified as potential endocrine disruptors, as they can mimic endogenous hormones, interfere with hormonal signaling, and act as agonists or antagonists of nuclear receptors. Through these mechanisms, endocrine-disrupting chemicals may alter hormone synthesis, transport, metabolism, and biological action. According to the United States Environmental Protection Agency (EPA), an endocrine-disrupting compound is defined as any exogenous agent that interferes with the synthesis, secretion, transport, binding, or elimination of natural hormones responsible for the maintenance of homeostasis, reproduction, development, or behavior (1).

Di(2-ethylhexyl) phthalate (DEHP) is a high–molecular weight plasticizer widely used in industrial applications to confer flexibility and malleability to polymeric materials, thereby increasing their durability and useful life. However, because DEHP is not covalently bound to the polymer matrix, it can leach over time into the surrounding environment or migrate into materials with which it comes into contact. DEHP is commonly found in personal care products, medical devices, cosmetics, toys, and food packaging; consequently, oral ingestion represents one of the primary routes of human exposure (2).

Accumulating evidence indicates that DEHP acts as an endocrine disruptor and has been associated with the development of metabolic disorders, including diabetes, hypertension, increased adiposity, dyslipidemia, and obesity (3, 4). In addition, DEHP exposure has been linked to endocrine alterations such as thyroid dysfunction and impaired sexual development in both males and females (5).

The life stage during which exposure to external agents, such as DEHP, occurs is crucial, with developmental windows (critical periods) of greater susceptibility to environmental influences (6). The first thousand days of life and the perinatal and postnatal periods are vital for metabolic programming and tissue differentiation. Exposure to environmental stressors during these phases can cause persistent physiological alterations, increasing susceptibility to non-communicable diseases in adulthood, such as obesity, diabetes, and cardiovascular disorders (7). These metabolic disruptions, which can remain latent for years, are the focus of the DOHaD hypothesis (Developmental Origins of Health and Disease), which proposes that early exposures exert long-lasting effects on long-term health (8). Given the rise in obesity and metabolic disorders, it is essential to investigate the role of endocrine disruptors like DEHP, which can be transferred during lactation. Thus, elucidating how exposure to DEHP during lactation influences metabolic health in adulthood is crucial for developing effective prevention strategies.

Given the alarming rise in obesity across all age groups, it is imperative to investigate factors that may contribute to this trend. Among these, environmental endocrine disruptors such as DEHP are of particular concern, as they can cross the placental barrier during pregnancy and be transferred to offspring through breast milkduring lactation. Exposure to these compounds during critical developmental windows may have profound and lasting consequences for metabolic health. Therefore, elucidating the effects of DEHP exposure during lactation on health outcomes in adulthood is essential for the development of effective prevention and intervention strategies.

A hallmark of exposure to endocrine-disrupting chemicals is the presence of non-monotonic dose–response relationships, in which biological effects do not increase linearly with dose. These compounds are capable of interacting with multiple signaling pathways, and their effects may vary according to the concentration and timing of exposure. At specific doses, endocrine disruptors may activate or inhibit particular molecular pathways, while at others they may trigger compensatory mechanisms that partially attenuate their action. Consequently, increasing the dose does not necessarily result in a proportional increase in adverse outcomes; instead, distinct effects may arise at different doses or levels of biological organization that are not evident at lower exposures (9).

The No Observed Adverse Effect Level (NOAEL) for DEHP has been established at 4.8 mg/kg/day in rats (10), and the acceptable daily intake has been set at 50 μg/kg body weight/day (11). In hospitalized patients in Italy, exposure to DEHP via oral intake and medical devices resulted in an estimated daily intake of 3.1 ± 0.9 μg/kg body weight (12). A New Zealand study published in 2021 reported a combined concentration of three DEHP metabolites (MEHP + MEOHP + MEHHP) of 19.0 μg/L in adults and 37.0 μg/L in children, highlighting the increased susceptibility of the pediatric population. Evidence of human exposure to DEHP is not limited to the Global North. In the Global South, a Brazilian study estimated daily DEHP intake levels of 29.8 μg/kg body weight/day in children associated with the use of plastic toys (13).

In parallel with the global increase in obesity across different age groups, growing evidence suggests that early exposure to endocrine-disrupting chemicals may contribute to long-term metabolic dysfunction. Exposure to DEHP during sensitive developmental periods may interfere with hormonal signaling, adipose tissue development, and energy homeostasis, thereby predisposing individuals to obesity and related metabolic disorders in adulthood.

In this context, the study by Smerieri et al. (14) reported higher urinary levels of the DEHP metabolite mono(2-ethylhexyl) phthalate (MEHP) in obese childrencompared with normal-weight children (0.27 vs. 0.15 μg/g creatinine-adjusted urine). Thus, understanding how exposure to DEHP during lactation can influence health outcomes later in life is essential for elucidating disease mechanisms and for the development of effective prevention and intervention strategies within the DOHaD paradigm.

## Methods

### Ethical approval and animals

All experimental procedures were conducted in accordance with the guidelines of the Federal University of Rio de Janeiro Animal Research Ethics Committee and were approved under protocol number A13/23-099/19. Nine adult female Wistar rats (2 months and 15 days old) were maintained under standard laboratory conditions (22 ± 0.5 °C room temperature, 50–60% relative humidity, and a 12 h light/dark cycle), with free access to food and water. Animals were acclimatized for 14 days prior to the experimental procedures.

### Mating and experimental design

After acclimatization, nine adult females were randomly assigned for mating and monitored throughout pregnancy and lactation. Near parturition, dams were housed individually. After birth, the litters were standardized with six male pups per dam to minimize variability related to litter size.

**Figure 1:**
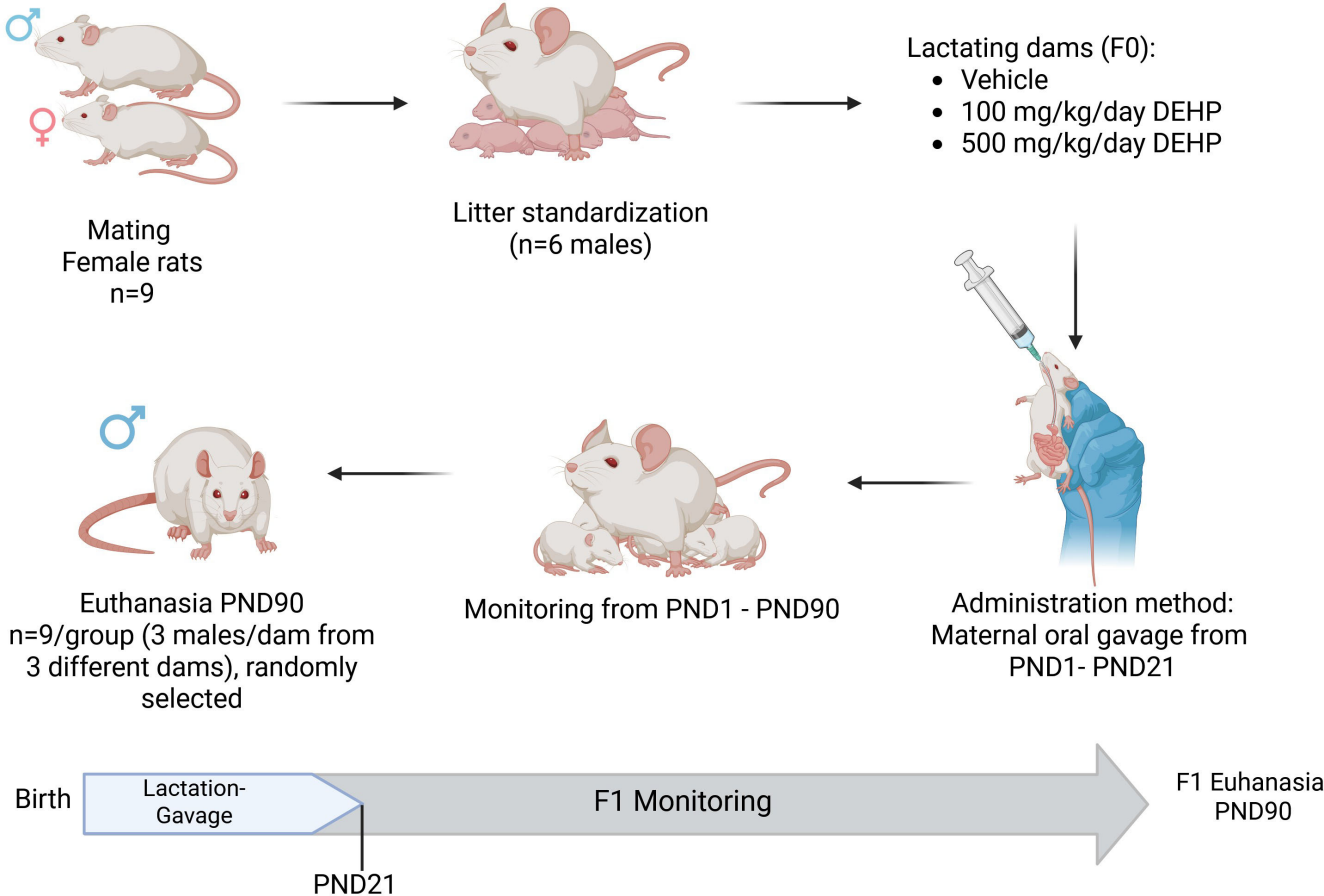
Schematic representation of the experimental design, including DEHP administration periods and sample collection protocol.

Dams were divided into 3 groups, 3 mothers per group, treated with di(2-ethylhexyl)phthalate (DEHP; CAS No. 117-81-7; purity ≥99.5%; Sigma-Aldrich, USA) dissolved in sesame oil (CAS No. 8008-74-0; Sigma-Aldrich, USA), or with vehicle only (sesame oil) as the control group. 0; Sigma-Aldrich, USA), or with vehicle only (sesame oil) as the control group. Treatments were administered once daily by maternal oral gavage from postnatal day (PND) 1 until weaning (PND21), totaling 21 consecutive days (7 days/week). The experimental groups were defined as follows (**Figure 1**):

- 500 DEHP group: 500 mg/kg body weight/day
- 100 DEHP group: 100 mg/kg body weight/day
- Control group: sesame oil only

The study doses of 100 mg/kg and 500 mg/kg are commonly employed in experimental toxicology models and correspond to Human Equivalent Doses (HED) of approximately 16 mg/kg/day and 81 mg/kg/day, respectively (15). These exposure levels are within the range reported for DEHP exposure associated with the use of medical devices, as described by Cambien et al. (16).

It should be noted that, in the present study, the treatments were administered to dams, and following maternal metabolism, DEHP or its metabolites were transferred to the offspring via lactation. Therefore, the actual internal dose received by the offspring is expected to be lower than the administered maternal dose. The absence of urinary biomonitoring data in the offspring constitutes a limitation of this study and will be addressed in future investigations to better characterize internal exposure levels.

### Offspring euthanasia

Male offspring were euthanized at weaning (PND21) or in adulthood (PND90), with nine animals per group per age (n = 9/group/age) randomly assigned. Each experimental group consisted of three offspring from each litter to ensure variability among groups.

Each pup in the litter was considered an independent experimental unit (N). Rodents exhibit social and physical hierarchy behavior, even as pups: larger and stronger individuals tend to suckle at the inguinal glands (usually associated with higher milk production) and for longer periods than smaller ones. Since DEHP is lipophilic, milk production and the volume of DEHP present can vary throughout the day, increasing the variability of exposure within the litter (17). The demand for milk production begins to be induced during gestation. Thus, adjusting the litters to an artificial number can affect the availability of milk for the animals (18, 19). Furthermore, maternal behavior may be differentiated within the litter, especially considering administration via gavage, a factor that can influence the behavior of lactating rats (20).

### Nutritional assessment and central adiposity

Body weight and food intake of the offspring were monitored weekly until PND90. At euthanasia, visceral white adipose tissue (vWAT) depots - including retroperitoneal, perigonadal, and mesenteric fat—were excised and weighed to assess central adiposity. The Lee index was calculated as an indicator of body composition using the following formula:

Lee index = weight (kg)1/3/nasoanal distance²) x 1000. This index is used to assess body composition and can help identify whether a person is within a healthy weight range in relation to their height.

### Assessment of glycemic homeostasis

Blood glucose was assessed in fasting animals (12 hours) using a glucometer (ACCU CHECK-Active, Roche^®^), using glucose oxidase test strips. The HOMA-IR and HOMA-β indices are parameters that serve to assess the insulin resistance index and the functional capacity of pancreatic β cells, respectively, and were calculated from the following data:

HOMA-IR = fasting insulin (MICRO UI/mL) x fasting glucose (mmol/L*)/22.5. HOMA- β = (20 x fasting insulin (MICRO UI/mL))/(fasting glucose (mmol/L*) - 3.5) (TANG et al., 2015).

### Radioimmunoassay

Serum levels of total triiodothyronine (T3) and thyroxine (T4) were determined using commercial radioimmunoassay kits (ImmuChem™ ^125I coated tube; ICN Biomedicals Inc., Orangeburg, NY, USA), following the manufacturer’s instructions.

### Enzyme-linked immunosorbent assay

Serum levels of insulin (E-EL-R2466.480), leptin (E-MSEL-M0033.480), and thyroid-stimulating hormone (TSH; E-EL-R0976) were quantified using enzyme-linked immunosorbent assay (ELISA) kits (Elabscience Biotechnology Inc., Wuhan, China), according to the manufacturer’s protocols.

For hormone-sensitive lipase (HSL) determination (HSL; E-EL-R2475), vWAT samples were processed by homogenizing 0.1 g of tissue in 1 mL of 0.01 M phosphate-buffered saline (PBS; pH 7.4), followed by centrifugation at 5, 000 × g for 10 minutes at 4 °C. The resulting supernatant was used for ELISA analysis (Elabscience Biotechnology Inc., China).

### Lipid profile assessment

Serum total cholesterol (TC) and triglyceride levels were measured using commercial colorimetric kits (Bioclin, Belo Horizonte, Brazil). Very-low-density lipoprotein cholesterol (VLDL-c) levels were estimated using the formula triglycerides/5 (mg/dL), as previously described (21).

### Protein extraction and western blot analysis

Total hypothalamus were used to protein extraction using lysis buffer composed of 50 mM Tris-HCl (pH 7.4), 150 mM NaCl, 1% Triton X-100, 0.1% SDS, 5 mM EDTA,

5 mM NaF, 0.69 mM sodium pyrophosphate, and 0.97 mM sodium orthovanadate, supplemented with 10% protease inhibitor cocktail (P8340; Sigma-Aldrich, St. Louis, MO, USA) to prevent protein degradation. Protein concentrations were determined using the bicinchoninic acid (BCA) assay (23227; Thermo Fisher Scientific, Waltham, MA, USA). Protein samples were mixed with 5× SDS-PAGE loading buffer and denatured by boiling for 5 minutes. Equal amounts of protein were separated on 12% SDS–polyacrylamide gels and subsequently transferred onto polyvinylidene difluoride (PVDF) membranes (Millipore, USA). Membranes were cut according to molecular weight markers and blocked with 5% nonfat dry milk diluted in Tris-buffered saline containing 0.1% Tween-20 (TBST) for 1 hour at room temperature with gentle agitation to prevent nonspecific binding. The membranes were incubated overnight at 4 °C with the following primary antibodies: proopiomelanocortin (POMC; 1:1000; 66358-1; Proteintech, USA), suppressor of cytokine signaling 3 (SOCS3; 1:1000; 66797-1; Proteintech, USA), neuropeptide Y (NPY; 1:1000; 12833-1-AP; Proteintech, USA), and β-actin (1:1000; 4970; Cell Signaling Technology, USA), which was used as a loading control. After primary antibody incubation, membranes were washed three times with TBST (5 minutes each) and incubated with the appropriate horseradish peroxidase (HRP)–conjugated secondary antibodies. Immunoreactive bands were visualized using an enhanced chemiluminescence detection system (RPN2232; Cytiva, USA). Images were acquired using the LAS 400 imaging system, and densitometric analysis was performed with ImageJ software.

### Statistical analysis

Data for all groups are expressed as mean ± standard error of the mean. One- way ANOVA followed by Bonferroni’s multiple comparisons test was performed using GraphPad Prism (version 8.0.1 for Windows 11, GraphPad Software, Boston, Massachusetts USA).

## Results

### Outcomes at weaning (PND21)

At weaning, offspring exposed to DEHP during lactation exhibited a significant reduction in body weight (53.33 ± 3.40 g, *p* < 0.05) and central adiposity (0.656 ± 0.122 g, *p* < 0.05) in the 500DEHP group compared to control group (**Table 1**). These animals also showed increased serum insulin levels (1.12 ± 0.48 mg/dL, *p* < 0.05), HOMA-β index (98.1 ± 45.06, *p* < 0.05), and total T3 levels (2.888 ± 0.686, *p* < 0.05).There were no statistical differences observed in the murimetric and tissue parameters, nor in the biochemical analyses of the group exposed to 100DEHP.

**Table 1:** Murinometric, tissue and related serum biochemical analyzes to PND21. Number of animals (n) = 5-7/group.

	Vehicle	100DEHP	500DEHP
Body weight (g)	62,44 ± 2,29	59,28 ± 3	53,33 ± 3,4*
Lee index	0,290 ± 0,019	0,293 ± 0,017	0,298 ± 0,014
Liver/BW	4,096 ± 1,085	4,083 ± 0,529	3,714 ± 0,482
WAT/BW	1,049 ± 0,294	0,845 ± 0,236	0,656 ± 0,122*
Insulin (mg/dL)	0,638 ± 0,068	0,715 ± 0,11	1,12 ± 0,483 *
Blood glucose (mg/dL)	89 ± 14,42	94,4 ± 8,96	91,1 ± 7,91
HOMA-IR	3,273 ± 0,665	3,727 ± 0,842	5,371 ± 2,275
HOMA-β	50,68 ± 9,67	55,65 ± 7,744	98,1 ± 45,06*
T3 (ng/mL)	0,499 ± 0,542	1,636 ± 0,634	2,888 ± 0,686
T4 (ng/mL)	6,342 ± 0,99	6,867 ± 1,81	8,179 ± 2,06
TSH (ng/mL)	44,7 ± 36,3	73 ± 47,73	16,7 ± 1,086
Leptin (ng/mL)	1,397 ± 0,421	2,744 ± 1,422	2,477 ± 0,957
HSL (ng/mL)	0,215 ± 0,0127	0,178 ± 0,033	0,209 ± 0,034

p<0.05, *difference in relation to the vehicle. HSL, hormone-sensitive lipase.

### Metabolic and adiposity outcomes in adulthood (PND90)

In adulthood, despite DEHP exposure during lactation, no significant differences were detected in body mass gain, final body mass, Lee index, or relative liver weight among the experimental groups. However, the 500DEHP group exhibited increased cumulative food intake over the 90-day period (2, 351 ± 33.01 g, *p* < 0.05) as well as a higher relative weight of visceral white adipose tissue (vWAT) (4.674 ± 1.614 g, *p* < 0.05) compared with controls (**Table 2**). The 100DEHP group did not show statistical differences in the parameters of weight, food intake, and analyzed tissue weights.

**Table 2:** Murinometric and relative tissue analyzes at PND90. Number of animals (n) = 4-7/group.

	Vehicle	100DEHP	500DEHP
Body weight (g)	429,65 ± 40,24	417,3 ± 48,26	486,55 ± 59,88
Total intake food (g)	2367,8 ± 200,6	2376,4 ± 215,2	2506,8 ± 67,6*
Lee index	0,299 ± 0,014	0,303 ± 0,005	0,304 ± 0,007
Liver/BW	3,13 ± 0,44	2,91 ± 0,16	3,19 ± 0,32
WAT/BW	3,7 ± 0,912	2,88 ± 0,379	4,48 ± 1,61*

p<0.05, *difference in relation to the vehicle.

### Endocrine outcomes in adulthood (PND90)

**Figure 2:**
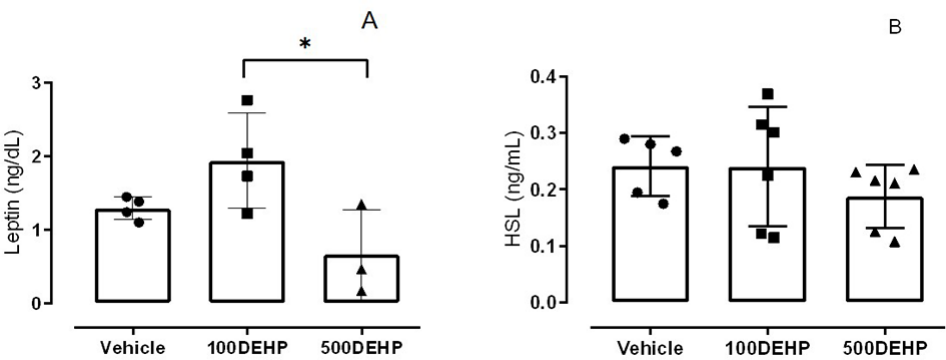
Hormone levels assessed by ELISA at postnatal day 90 (PND90). Serum levels of leptin **(a)** and vWAT content of hormone-sensitive lipase (HSL) **(b)**. data were analyzed using one-way ANOVA followed by Bonferroni *post hoc* test. *p < 0.05 compared to the 100EHP group. Number of animals (n) = 3-6.

Serum leptin levels were significantly reduced in adult animals from the 500DEHP group (0.6598 ± 0.6112 ng/dL, *p* < 0.05) (**Figure 2a**). In contrast, no significant differences were observed in hormone-sensitive lipase (HSL) protein levels in vWAT between groups (**Figure 2b**).

**Figure 3:**
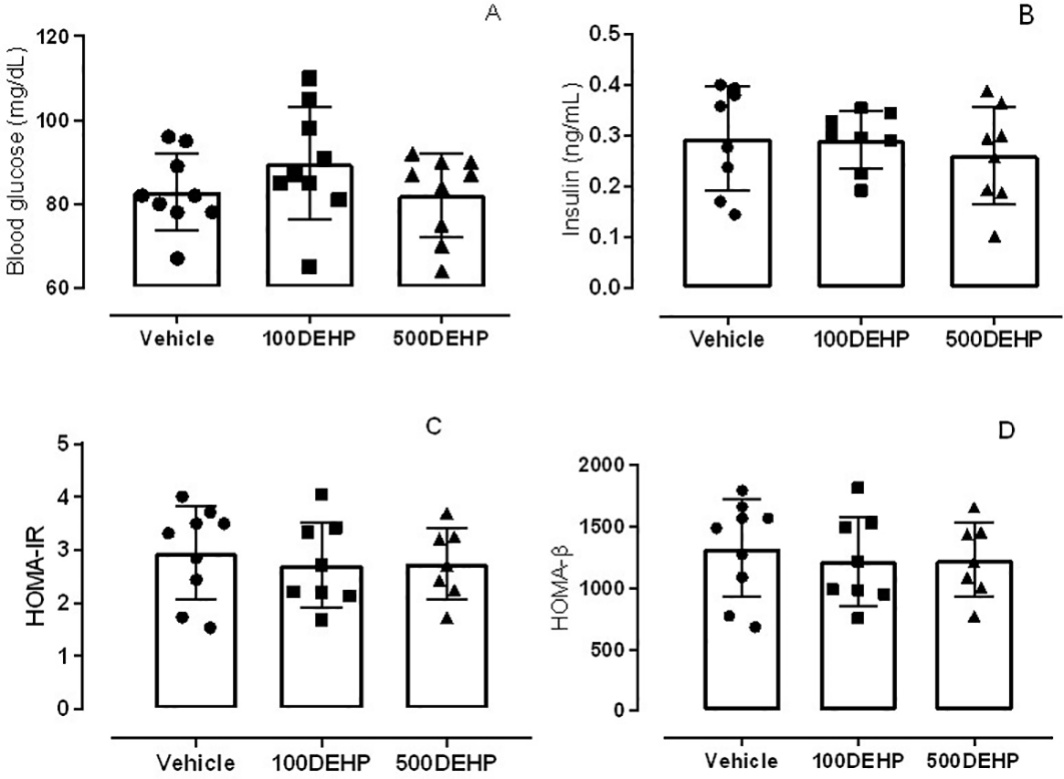
Glycemic homeostasis at postnatal day 90 (PND90). Fasting blood glucose **(a)**, serum insulin levels **(b)**, HOMA-IR **(c)**, and HOMA-β index **(d)**. data were analyzed using one-way ANOVA followed by Bonferroni *post hoc* test. Number of animals (n) = 7-9.

**Figure 4:**
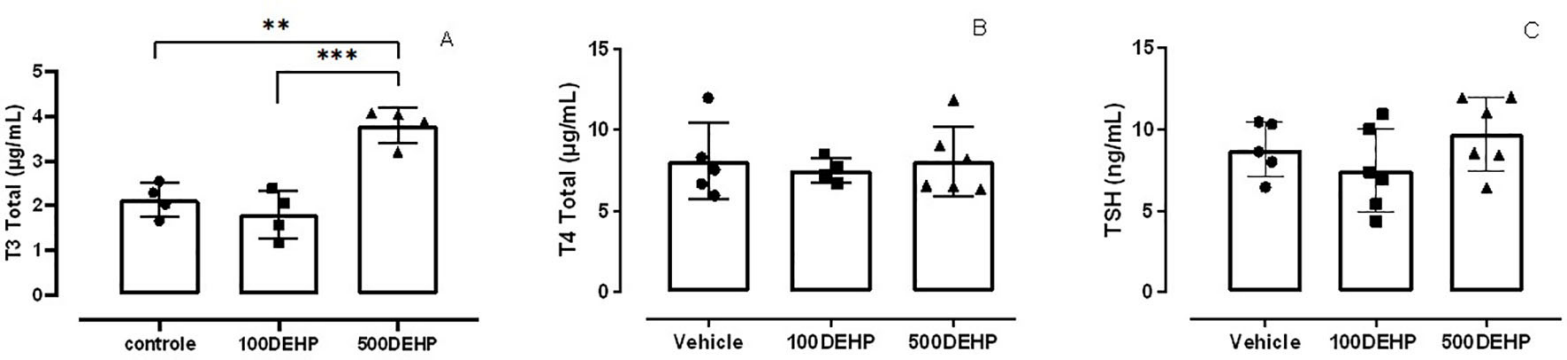
Thyroid function at postnatal day 90 (PND90). Serum total T3 levels **(a)**, serum total T4 levels **(b)**, and serum TSH levels **(c)**. data were analyzed using one- way ANOVA followed by Bonferroni *post hoc* test. **p<0.01 500DEHP *vs* sesame oil vehicle group, ***p<0.001 500DEHP *vs* 100DEHP group. Number of animals (n) = 4- 6.

Fasting blood glucose (**Figure 3a**) and serum insulin levels (**Figure 3b**) did not differ significantly among groups. Similarly, no differences were observed in HOMA-IR (**Figure 3c**) or HOMA-β (**Figure 3d**) indices.Adult animals from the 500DEHP compared to the control group showed increased serum total T3 levels (2.868 ± 0.678, *p* < 0.05) (**Figure 4a**), whereas no significant differences were observed in total T4 or TSH levels among groups at 90 days of age (**Figures 4b, c**).

**Figure 5:**
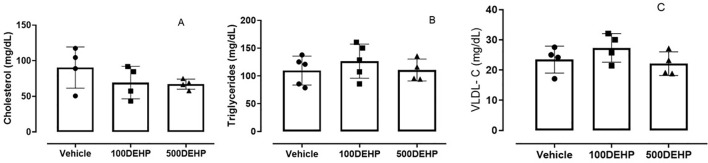
Serum lipid profile at postnatal day 90 (PND90). Total cholesterol **(a)**, triglycerides **(b)**, and VLDL-C levels **(c)**. data were analyzed using one-way ANOVA followed by Bonferroni *post hoc* test. Number of animals (n) = 4-5.

No significant alterations were detected in the serum lipid profile, including cholesterol, triglycerides, and VLDL-c levels, in adult male rats exposed to DEHP during lactation (**Figure 5**).

### Hypothalamic protein expression (PND90)

**Figure 6:**
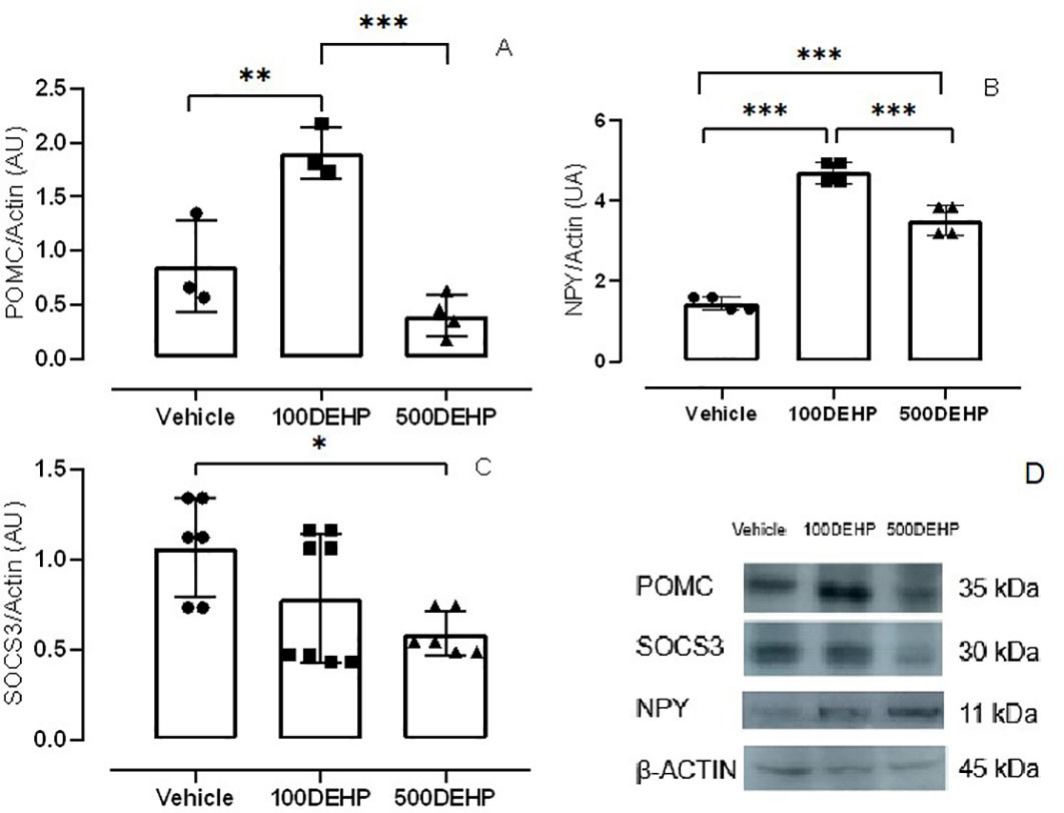
Hypothalamic proteins involved in the regulation of food intake at postnatal day 90 (PND90) analyzed by Western blotting. Protein expression levels of **(A)** POMC, **(B)** NPY, and **(C)** SOCS3, and **(D)** their respective representative bands. Data were analyzed using one-way ANOVA followed by Bonferroni *post hoc* test. *p < 0.05, **p < 0.01, and ***p < 0.001 indicates a significant difference between groups. Number of animals (n) = 3–4.

In the hypothalamus, the 500DEHP group exhibited reduced protein expression of POMC (0.4740 ± 0.1398, *p* < 0.01) and SOCS3 (0.5887 ± 0.1202, *p* < 0.05), accompanied by increased NPY protein expression (3.508 ± 0.4658, *p* < 0.05) (**Figure 6**). In contrast, the 100DEHP group showed a different profile, characterized by a simultaneous increase in both POMC (1.905 ± 0.2372) and NPY protein expression (4.695 ± 0.3288, *p* < 0.001) compared with the control group. This dose-dependent profile, with distinct hypothalamic responses for each concentration, evidences the non-monotonic action of the endocrine disruptor DEHP.

## Discussion

The increasing prevalence of obesity and metabolic disturbances worldwide is a multifactorial phenomenon, strongly linked to lifestyle changes and widespread exposure to environmental chemicals such as phthalates.

**Figure 7:**
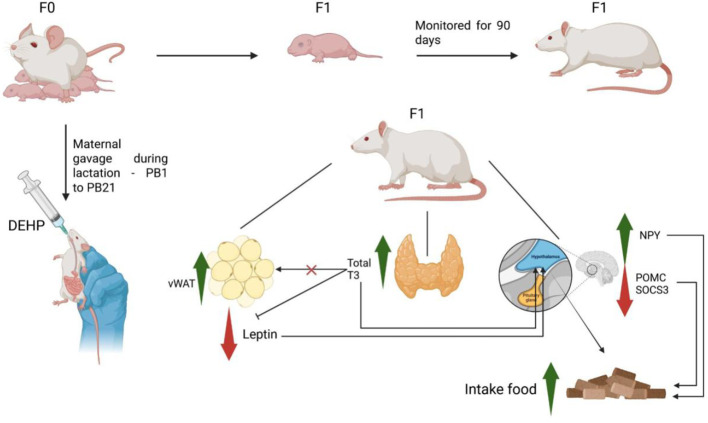
Summary of the main neuroendocrine and metabolic findings of the study.

In this context, the present study modeled exposure to DEHP during lactation in Wistar rats, a crucial developmental period, to elucidate the mechanisms through which lactational exposure to DEHP may influence metabolic development and health later in life (**Figure 7**).

At postnatal day 21, offspring from dams exposed to 500 mg/kg body weight/day of DEHP exhibited reduced body weight and central adiposity, consistent with previous findings (22). In contrast, Strakovsky et al. reported that male offspring exposed to 300 mg/kg/day of DEHP during gestation and lactation displayed reduced adiposity at birth followed by increased adipogenesis during lactation. Although these findings differ from those observed in the present study, they reinforce the ability of DEHP to modulate adipogenesis and body weight, suggesting that the timing of exposure critically determines the adipogenic outcome. DEHP exposure during this critical period resulted in growth restriction and adiposity in the animals at weaning. This early compromise may have the potential to affect health and contribute to metabolic disorders in adult life.

Regarding glycemic parameters at weaning, fasting blood glucose levels were unaltered; however, offspring in the 500DEHP group exhibited increased serum insulin levels and HOMA-β index, indicating enhanced pancreatic insulin secretion and a possible early-stage desensitization to insulin signaling. These findings are partially divergent from those reported by Rajesh and Balasubramanian (23), who demonstrated reduced insulin secretion, hyperglycemia, and pancreatic dysfunction in offspring exposed to DEHP during gestation. Similarly, other studies using DEHP exposure during gestation and lactation, with doses of 1.25 and 6.25 mg/kg/day, demonstrated short-term effects in Wistar rats. A reduction in glycemia and insulin levels was observed, as well as a reduction in the oral glucose tolerance test curve. These findings suggest that the effects of DEHP may vary depending on whether the analysis is short-term or long-term (24). Such discrepancies suggest that both the duration and timing of DEHP exposure are critical determinants of its effects on pancreatic function and glycemic homeostasis. At 90 days of age, animals in the 500DEHP group exhibited increased cumulative food intake and vWAT mass without changes in total body weight, suggesting alterations in body composition.

Despite these alterations, no disruption of glycemic homeostasis was observed in adult offspring, as evidenced by normal fasting glucose, insulin levels, and HOMA indices. This suggests that the hyperinsulinemic profile observed at weaning may be a transient effect, which is consistent with metabolic adaptation following cessation of DEHP exposure, given its restriction to the lactation period.

Serum total T3 levels were elevated at both developmental stages in animals exposed to DEHP, while T4 and TSH levels remained unchanged. The increase in T3 at weaning may reflect DEHP-induced modulation of thyroid hormone synthesis or peripheral conversion, potentially influencing energy expenditure during this critical developmental window. The persistence of elevated T3 levels until adulthood, in the absence of changes in T4 or TSH, suggests long-term alterations in thyroid hormone metabolism, possibly involving deiodinase activity.

Although direct evidence of increased T3 levels following DEHP exposure is limited, similar effects have been reported with other phthalates. For example, gestational exposure to di-n-hexyl phthalate resulted in increased T3 levels in male offspring (25). Previous studies have also demonstrated that DEHP alters the expression of deiodinases. Changes in deiodinase mRNA levels have been reported in adolescent and adult rats following subchronic or chronic DEHP exposure (26, 27). Thus, the permanent elevation in T3 levels may be consistent with previous findings that DEHP alters deiodinase activity or alternatively, this may reflect an early effect of neonatal exposure to DEHP on the regulation of the hypothalamic–pituitary–thyroid axis, leading to permanent impairment of the axis and its hormone production. These hypotheses will be tested in future studies.

No significant changes were detected in the serum lipid profile of adult animals. In contrast, prenatal DEHP exposure has been associated with dyslipidemia in other models (28), suggesting that the lactation period may be less sensitive to DEHP-induced alterations in lipid metabolism.

A significant reduction in serum leptin levels was observed in adult animals exposed to DEHP during lactation. This finding contrasts with previous studies reporting increased leptin levels following gestational or chronic DEHP exposure (29, 30). The divergence may reflect differences in exposure windows, as the lactation period has not been previously examined in isolation. It is possible that perinatal exposure to DEHP during lactation selectively impairs leptin synthesis or secretion by adipose tissue, with long-term metabolic consequences.

At the central level, hypothalamic alterations observed in the 500DEHP group provide mechanistic insight into the adult metabolic phenotype. Neonatal exposure to high-dose DEHP programmed an obesogenic phenotype characterized by hyperphagia, and higher visceral adiposity, which could be linked to impaired leptin signaling originating from adipose tissue dysfunction. This hypoleptinemia was accompanied by reduced hypothalamic POMC and increased NPY protein expression evidencing a central response to the lower level hormonal of leptin. Reduced SOCS3 expression may indicate a maintained hypothalamic sensitivity, enabling the central nervous system to respond to a peripheral “false hunger” signal generated by reduced leptin availability.

Previous studies have suggested that thyroid hormones may modulate hypothalamic regulation of energy balance. In deiodinase 3 knockout mice, increased T3 levels were associated with elevated NPY and reduced POMC and SOCS3 expression (31). While leptin levels were not reduced in young animals in that model, older females exhibited decreased leptin levels, partially aligning with our findings. Nevertheless, in the present study, increased adiposity accompanied by reduced leptin secretion suggests a primary dysfunction of adipose tissue rather than impaired hypothalamic responsiveness.

Interestingly, the 100DEHP group exhibited a distinct hypothalamic response, characterized by increased POMC and NPY expression, suggesting a non-linear, dose-dependent effect of DEHP on hypothalamic neurocircuitry. As these pathways exert opposing roles in the regulation of energy homeostasis and food intake, the simultaneous upregulation of both anorexigenic and orexigenic signals could represent a complex, yet undetermined, state of central dysregulation or a dose-specific compensatory mechanism. This biphasic pattern is consistent with the non-monotonic dose–response behavior commonly described for endocrine-disrupting chemicals and underscores the complexity of DEHP action on central metabolic regulation.

In conclusion, the lactation period represents a critical but underexplored window in DOHaD research. Our findings demonstrate that neonatal exposure to DEHP via breast milk induces metabolic programming effects in male offspring, characterized by transient alterations in glycemic homeostasis and persistent changes in adiposity, leptin signaling, and thyroid hormone metabolism. These long-term neuroendocrine alterations occur even in the absence of overt metabolic disease, reinforcing the importance of minimizing exposure to endocrine-disrupting chemicals during early development to promote lifelong metabolic health.

## Data Availability

The original contributions presented in the study are included in the article/supplementary material. Further inquiries can be directed to the corresponding author.
