# Supplementary figures and images for: Neonatal exposure to di-(2-ethylhexyl) phthalate (DEHP) through breastfeeding leads to dysfunction endocrine-metabolic outcomes in male rats at adulthood
Source: Front Endocrinol (Lausanne). 2026 Jun 3;17:1826776. doi: 10.3389/fendo.2026.1826776 (PMC13271919; doi:10.3389/fendo.2026.1826776)

Uncropped Figure 5D:

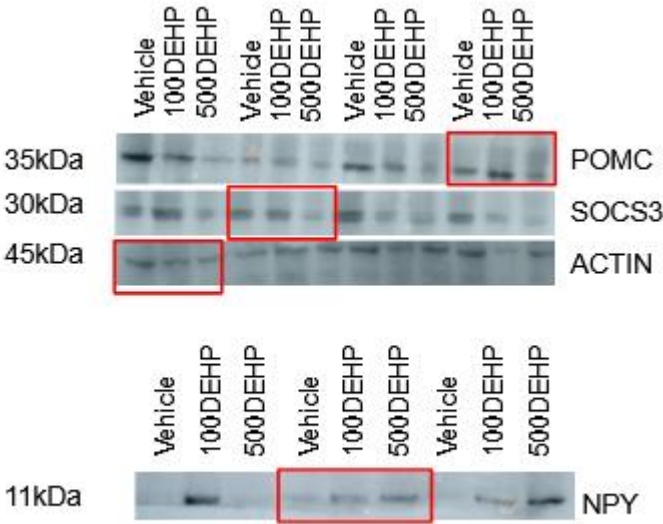

Supplementary:

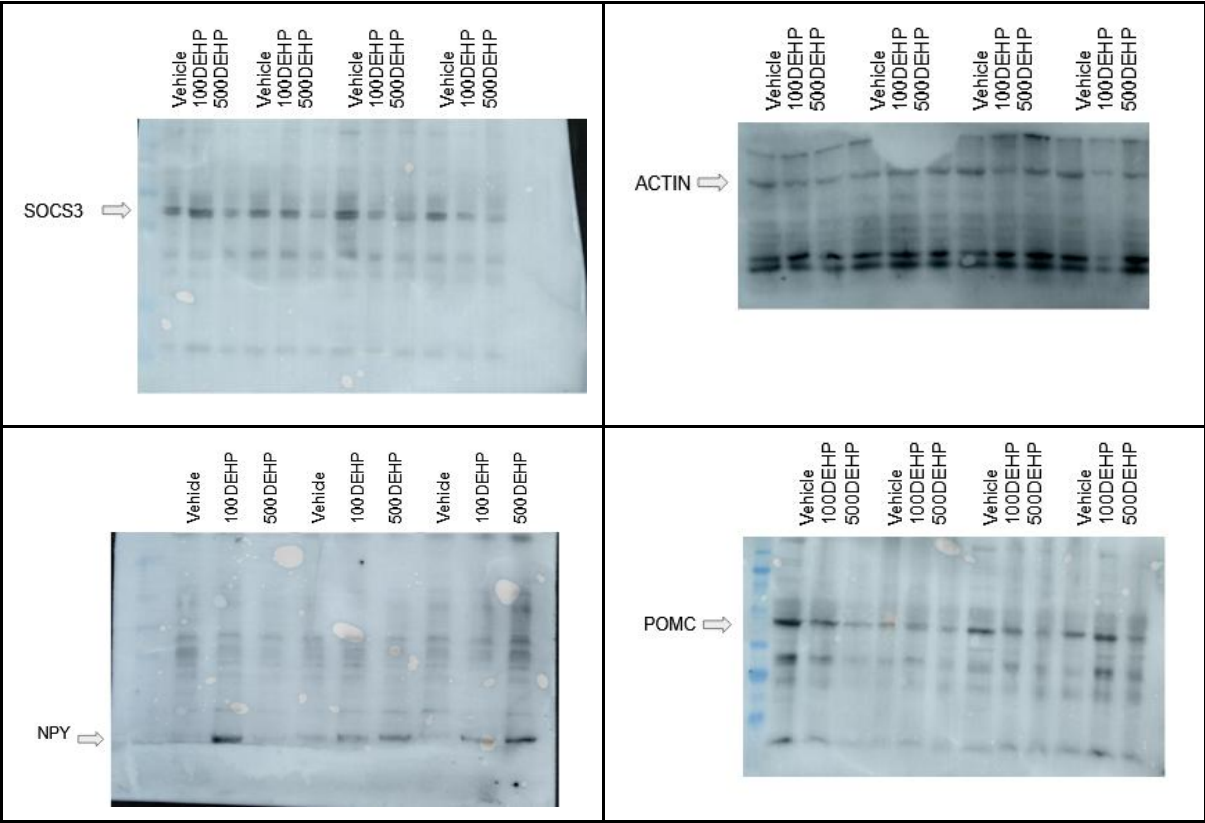

Supplement: Supplementary file 1 [file DataSheet1.pdf]
